# A new method for accurate assessment of DNA quality after bisulfite treatment

**DOI:** 10.1093/nar/gku904

**Published:** 2014-10-11

**Authors:** M. Ehrich, S. Zoll, S. Sur, D. van den Boom

Nucl. Acids Res. (2007) 35 (5): e29 doi: 10.1093/nar/gkl1134

The panels were inadvertently duplicated in Figure 5. A new Figure 5 is included, where the lower panels have been replaced with the original data. This error does not affect the results or the conclusion of the article. The authors apologize to the readers and publisher for any inconvenience this may have caused.

**Figure 5.**
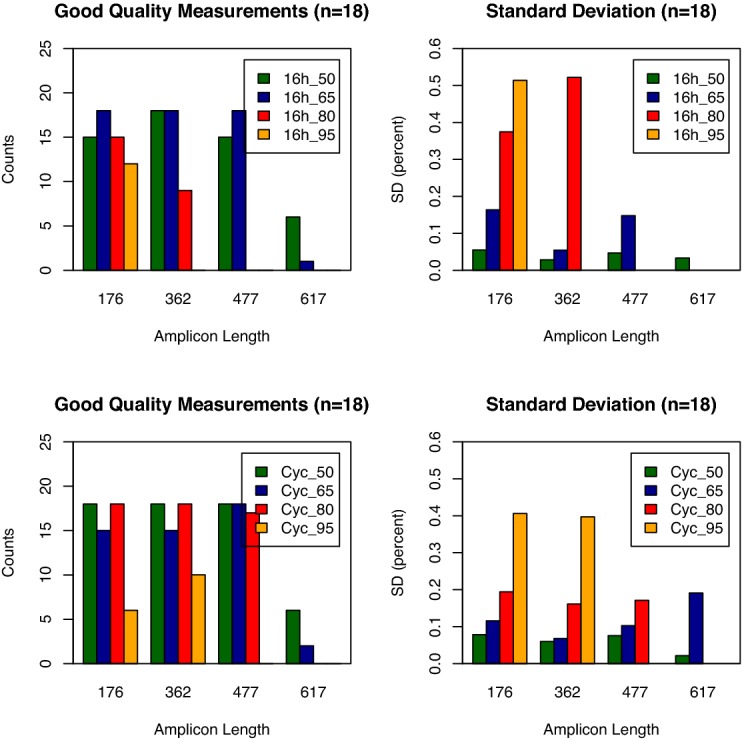
Bar graphs showing the number of high quality mass spectra for each amplicon length (two panels on the left). The panels on the right side show the corresponding standard deviations of the quantitative measurements. The bar graphs show results for different bisulfite incubation protocols. The results from 16 h incubation at constant temperature are shown in the upper two panels and results from a cycled incubation protocol are shown in the lower two panels. A total of 18 reactions were performed for each amplicon. Cycled incubation and lower incubation temperatures result in higher amplification success for longer amplicons and lower standard deviations on the determination of methylation ratios.

